# Psychological Well-Being, Prenatal Attachment, and Quality of Early Mother-Infant Interaction: A Pilot Study With a Sample of Mothers With or Without Cancer History

**DOI:** 10.3389/fpsyg.2022.913482

**Published:** 2022-06-10

**Authors:** Laura Bozicevic, Lucia Ponti, Martina Smorti, Gabriella Pravettoni, Fedro Alessandro Peccatori, Chiara Cassani, Giuseppe Nastasi, Valentina Sarchi, Lucia Bonassi

**Affiliations:** ^1^Department of Primary Care and Mental Health, University of Liverpool, Liverpool, United Kingdom; ^2^Dipartimento di Studi Umanistici, Università di Urbino Carlo Bo, Urbino, Italy; ^3^Dipartimento di Patologia Chirurgica, Medica, Molecolare e dell’Area Critica, Università di Pisa, Pisa, Italy; ^4^Divisione di Psicologia, European Institute of Oncology IRCCS, Milan, Italy; ^5^Gynecologic Oncology Department, European Institute of Oncology IRCCS, Milan, Italy; ^6^Dipartimento di Ginecologia e Ostetricia, Fondazione IRCCS Policlinico San Matteo, Pavia, Italy; ^7^Oncologia Medica, ASST Bergamo Est, Seriate, Italy; ^8^Dipartimento Medico, ASST Bergamo Est, Seriate, Italy

**Keywords:** mother–infant interaction, cancer, prenatal attachment, maternal well-being, observational method

## Abstract

Given the positive impact of high-quality mother–infant interaction on child development, and that such relationship might be hindered by maternal stresses such past cancer, research is needed to understand protective and risk factors in this clinical population. As almost no data is available on the impact of history of cancer on the quality of mother–infant interaction, a multicentric and longitudinal pilot study was conducted. Differences in women’s prenatal psychological well-being and attachment (T1, third trimester), and postnatal quality of mother–infant interaction (T2, 2–5 months) were assessed in a sample of Italian mothers with (*N* = 11) or without cancer history (*N* = 13). Results showed that women did not differ significantly in their prenatal well-being (assessed with the Profile of Mood States questionnaire) and levels of attachment (assessed with the Prenatal Attachment Inventory). Looking at mother–infant interactions (assessed using the Global Rating Scale at T2), while maternal sensitivity, warmth and intrusiveness, and infant distress and attentiveness did not differ between the two groups, in the clinical group, mothers were more remote and less absorbed in the infant, and infants showed fewer positive communications. These findings might shed light on potential protective and risk factors for early parenting and later child outcomes in this clinical population.

## Introduction

Research demonstrates that a supportive caregiver-child relationship is essential for promoting optimal child socio-emotional and cognitive development (Rocha et al., 2020) and that parenting programmes designed to foster, for instance, sensitive and reciprocal mother–infant interaction can positively influence child outcomes (Britto et al., 2017). There is a wealth of literature showing that maternal sensitivity (i.e., the ability to appropriately and contingently detect and respond to infant’s signals), warmth (i.e., affectionate exchanges between mothers and their infants), and low demanding, critical and intrusive maternal behaviours contribute to create a safe and psychologically secure environment where children can learn about their emotions, be cognitively stimulated, and develop social skills (Petersen et al., 2017; Deans, 2020). However, risk factors such as poor maternal psychological well-being (e.g., depression and anxiety), can hinder the quality of such interactions and, consequently, be considered a risk factor for maladjusted child development (Lefkovics et al., 2014; Rogers et al., 2020). In fact, research demonstrated that poor mental health might have a negative impact on mother’s ability to bond with her infant both in pregnancy and postnatally, resulting in difficult interactions with her child (i.e., high levels of maternal intrusiveness, and low involvement and warmth; infant’s high distress, and low engagement and positive communication) (Shin et al., 2006; Heinisch et al., 2019; Hazell Raine et al., 2020).

Having a serious disease can be a risk factor for mental health. Specifically, cancer diagnosis during the transition to motherhood has a strong negative impact on maternal wellbeing (Schmitt et al., 2010; Mascheroni et al., 2020). However, to date, there is paucity of data on the impact of having received a diagnosis of cancer prior to pregnancy on the quality of mother–infant interaction; the few existing studies which include aspects related to children mainly focus on women with cancer during pregnancy and the side-effects of treatments administrated prenatally, such as on child cognitive or cardiovascular disease (Vandenbroucke et al., 2020), but not on the impact of the diagnosis on the developing relationship between mothers and infants nor on long-term effects of cancer diagnosis prior to pregnancy on such relationship.

Women with past cancer diagnosis seem to perceive pregnancy as a positive event in their life representing the possibility to go beyond the experience of the illness (Faccio et al., 2020; Mascheroni et al., 2020). However, these women seem to show low levels of prenatal attachment (Mascheroni et al., 2020) suggesting that they might find difficult to create a mental space for a relationship with their unborn child as they are still elaborating their experience of illness. Therefore, after birth, these mothers might encounter difficulties in establishing a positive mother-child relationship; research exploring these aspects is needed to better support these women in their process of adjustment to motherhood, and to prevent negative consequences of possible maternal difficulties on child development.

The aim of our study was to assess the psychological well-being and prenatal attachment in women with or without history of diagnosis of cancer and to compare the quality of mother–infant interaction at 2–5 months post-partum between the two groups of women.

## Methods

### Procedure and Participants

A multicentric and longitudinal pilot study was designed to assess group differences between mothers with past cancer diagnosis (with at least 5 years from complete remission in line with AIOM guidelines – clinical group – 11 women) and mothers without cancer (control group – 13 women) in their mental well-being, prenatal attachment and relationship with their infant. Women were recruited at the ASST Bergamo Est Hospital, IEO and IRCCS “San Matteo” Hospital between 2018 and 2019. Exclusion criteria included non-Italian speakers, and women with medical complications in pregnancy or foetus with serious genetic conditions or congenital abnormalities.

Demographic data, women’s mental well-being and prenatal attachment were collected during the third trimester of pregnancy (T1), while quality of mother–infant interaction was assessed at 2–5 months post-partum (T2). The study was conducted according to the guidelines for ethical treatment of human participants of the Italian Psychological Association and was approved by the ethical committee of the local health authorities (196/2016); before data collection, all women provided informed consent.

### Measures

Socio-demographical data gathered at T1 are described in Table 1 and included maternal age, education, marital and work status, parity, presence of previous miscarriages, type of pregnancy, and type of conception method. Additionally, women completed the Italian version of the Prenatal Attachment Inventory – PAI (Della Vedova et al., 2008) and the Italian version of the Profile of Mood States – POMS (Farnè et al., 1991). The PAI is a self-report questionnaire to assess women’s prenatal attachment to their foetus which includes 21 items rated on 4-point Likert scales. Higher scores indicate higher levels of foetal attachment. For the present sample, Cronbach’s alpha was.82. The POMS is a self-report questionnaire to assess mental well-being which includes 58 different feelings rated by participants on a 5-point Likert scale. Six mood-state scales are derived from the scores: tension-anxiety, depression, anger-hostility, vigour, fatigue, and confusion. Higher scores indicated worse mood or emotions in all scales but in the vigour one where higher scores indicated better mood or emotions. In the present sample, the Cronbach’s alpha coefficients ranged from .62 to .88.

**TABLE 1:** Socio-demographic characteristics by groups.

	Clinical group (*n* = 11)	Control group (*n* = 13)	*P*
*Age M* (SD)	37.36 (5.10)	33.92 (3.52)	0.065^a^
*Marital status n* (%)			0.932^b^
Married/Cohabiting	10 (90.9%)	13 (100%)	
Separated/divorced	1 (9.1%)	0 (0.0%)	
*Years of schooling M* (SD)	13.64 (4.11)	15.46 (2.40)	0.266^a^
*Work status n* (%)			0.278^b^
Precarious	0 (0.0%)	3 (23.1%)	
Stable	11 (100%)	10 (76.9%)	
*Parity n (%)*			0.039^b^
Primiparous	5 (45.5%)	12 (92.3%)	
Multiparous	6 (54.5%)	1 (7.7%)	
*Presence of previous miscarriages n* (%)			0.464^b^
Yes	3 (27.3%)	1 (7.7%)	
No	8 (72.7%)	12 (92.3%)	
*Type of pregnancy n (%)*			0.932^b^
Single	10 (90.9%)	13 (100.0%)	
Twin	1 (9.1%)	0 (0.0%)	
*Conception n* (%)			1.000^b^
Spontaneous conception	10 (90.9%)	12 (92.3%)	
Medically assisted procreation	1 (9.1%)	1 (7.7%)	

*^a^Mann–Whitney U test.*

*^b^Chi square test with Yates’ correction for continuity.*

At T2, mother–infant face-to-face interaction was video-recorded and its quality was assessed using the Global Rating Scale – GRS (Murray et al., 1996); trained coders rated the videos on the following maternal and infant scales: maternal sensitivity, warmth, intrusiveness, remoteness, and absorption in the infant, and infant attention to mother and to the environment, positive vocalisations and distress. Higher scores indicate better quality of the interaction (e.g., more sensitive and less intrusive mothers, more attentive and less distressed infants).

### Statistical Analyses

Mann–Whitney *U* tests or Chi Square tests with Yates’ correction for continuity were performed, depending on the dichotomous or continuous nature of variables, to compare the two groups on socio-demographical data and on all dependent variables (PAI score, dimensions of the POMS and the GRS scales) with a significance level p < 0.05.

## Results

A total of 24 pregnant women aged 27–48 (*M* = 35.36; SD = 4.53) were recruited for the present study, 11 (45.8%) in the clinical group, and 13 (54.2%) in the control group. Participants’ socio-demographical and clinic characteristics are presented in Table 1. In the clinical group, three women had had gynaecological cancer, six had breast cancer, and two had skin cancer. All of them had at least one treatment among chemotherapy, radiotherapy, surgical intervention.

### Group Differences

No significant differences were observed between the two groups in terms of their socio-demographic characteristics, except for parity (higher prevalence of primiparous in the control group – Table 1).

Groups did not differ significantly in their levels of prenatal attachment and their psychological profile, but significant differences emerged in some of the scales used to assess the quality of mother–infant interaction (Table 2); while dimensions such as maternal sensitivity, warmth and intrusiveness, and infant distress and attentiveness did not differ between the two groups, mothers from the clinical group were more remote and less absorbed in the infant, and their infants showed fewer positive communications compared to the control group (see Table 2).

**TABLE 2:** Statistical comparison between groups on PAI scores, POMS dimensions and GRS scales.

	Clinical group (*n* = 11)		Control group (*n* = 13)			
	Mean ranks	Sum of ranks	Mean ranks	Sum of ranks	*U*	*P*
PAI total score	12.45	137.00	12.54	163.00	71.00	0.977
POMS tension-anxiety	13.80	138.00	10.62	138.00	47.00	0.261
POMS depression	13.00	130.00	11.23	146.00	55.00	0.531
POMS anger-hostility	12.65	126.50	11.50	149.50	58.50	0.683
POMS vigour	11.80	118.00	12.15	158.00	63.00	0.901
POMS fatigue	12.10	121.00	11.92	155.00	64.00	0.950
POMS confusion	12.65	126.50	11.50	149.50	58.50	0.685
GRS (M) sensitive – insensitive	10.41	114.50	14.27	185.50	48.50	0.110
GRS (M) warm – cold	11.50	126.50	13.35	173.50	60.50	0.422
GRS (M) non-intrusive – intrusive	11.82	130.00	13.08	170.00	64.00	0.646
GRS (M) non-remote – remote	9.18	101.00	15.31	199.00	35.00	0.019
GRS (M) absorbed in the infant – self-absorbed	9.45	104.00	15.08	196.00	38.00	0.024
GRS (I) attentive – avoidant	10.27	113.00	14.38	187.00	47.00	0.099
GRS (I) engaged with the environment – self-absorbed	10.32	113.50	14.35	186.50	47.50	0.113
GRS (I) positive vocalisations – No positive vocalisations	8.64	95.00	15.77	205.00	29.00	0.010
GRS (I) happy – distressed distress	11.77	129.50	13.12	170.50	63.50	0.616

*PAI – Prenatal attachment.*

*POMS – Mental well-being.*

*GRS (M) – Quality of the mother–infant interaction (maternal scales).*

*GRS (I) – Quality of the mother–infant interaction (infant scales).*

## Discussion

The present longitudinal study builds on research evidence showing that the first postnatal months are highly sensitive for the development of the parent-infant relationship. New mothers face various challenges in their transition to parenthood including adjusting to new sleep patterns, managing feeding difficulties and learning how to respond to infant’s distress (Mascheroni et al., 2020). It is not surprising that additional stresses, such as an disease as cancer, might burden parents even more than a typical transition to parenthood does, and negatively impact on the newly developing relationship with their infants. This study was designed to explore maternal well-being, prenatal attachment and quality of mother–infant relationship in a sample of mothers with a diagnosis of cancer comparing them to a control group of mothers without cancer.

From our results it emerged that, in pregnancy, women in the clinical group did not experience higher psychological stress (e.g., anxiety, depression, anger); this is partially in line with some existing studies on populations of survivors of cancer and of woman diagnosed with cancer during pregnancy which showed that, in these groups, only some aspects of their psychological health (e.g., cognition, sexual function, fatigue, anxiety) are impaired, but not others (e.g., depressive symptoms, negative feelings, positive feelings) (Bradley et al., 2006; Mitchell et al., 2013; Carreira et al., 2021); moreover this result is similar to the ones of research specifically on pregnant women with a past, rather than concurrent, diagnosis of cancer who did not seem to differ in their mood states from their controls (Mascheroni et al., 2020). Finally, longitudinal studies demonstrated that mental health symptoms shown by cancer survivors tend to improve over time becoming similar to those of the general population around one year after the diagnosis (Stover et al., 2014; Bantema-Joppe et al., 2015) which might further explain the absence of differences found.

Regarding prenatal attachment, women in the clinical sample were found to be similarly attached to their foetus compared to women who did not have cancer, differently from what found by previous research (Mascheroni et al., 2020). There is still too little research on this topic, but one possible explanation of our finding is that mothers with past diagnosis of cancer were investing positive emotions on being pregnant as reported by previous qualitative studies (Schmitt et al., 2010; Faccio et al., 2020), which might have buffered the negative effects of the health conditions on the investment on the future infant.

Finally, results on the quality of mother–infant interactions showed that mothers with a past diagnosis of cancer engaged with their infants similarly to mothers without cancer in aspects such as how contingently and attuned they responded to their infants (i.e., sensitivity), how they expressed their affection toward their children (i.e., warmth) and how well they followed the infants cues instead of engaging in behaviours which are intrusive for the infants (i.e., intrusiveness); their children were attentive to their mother and the environment, and happy during the interaction similarly to their control infants. However, mothers with past cancer showed behaviours such as difficulties to remain engaged with their infants (i.e., higher remoteness) and being self-centred (i.e., low absorption in the infant) more often compared to women in the control group, and their infants were less communicative (i.e., showing fewer positive vocalisations). Such maternal behaviours might be a defensive way to avoid getting attached too soon to their infants in mothers who experienced uncertainties about their health, or they might be the expression of psychological difficulties emerging in the postpartum, as in pregnancy the two group of mothers did not differ in their well-being. It is possible that the difficulties found in mothers negatively influenced the infant vocal behaviours, which was poorer in the clinical group compared to the controls, even though this hypothesis could not to be tested as the two behaviours were assessed at the same time-point. Similarly, previous studies showed that, independently from possible maternal depression, a withdrawn and self-absorbed maternal style of interaction is associated with concurrent lower infants’ activity and communication (Gunning et al., 2004; Costa and Figueiredo, 2012). Further research is needed to determine long-term effects of these disruptions in early mother–infant interactions on later child socio-emotional development in samples of mothers with past diagnosis of cancer.

### Study Strengths and Limitations

The main limitation of this research is the small sample size, so results should be interpreted with caution and future research with larger samples is needed to replicate the study. Moreover, maternal antenatal attachment and mood were assessed using self-report measures which might be biased; therefore, future research could employ different kind of tools, such as diagnostic interviews done by a clinician to assess these dimensions. Along with these limitations, this study presents some strengths: the sample was homogeneous due to the strict inclusion criteria for clinical group, the study design was longitudinal and not retrospective which guaranteed a better accuracy of the data collected, and the measure used to qualify the mother–infant interaction was observational which implies that the coding from trained assessors is independent from mothers’ willing to report and awareness of certain aspects of their relationship with their infants, therefore it is more accurate.

### Implications for Practice and/or Further Research

On one hand, our findings might suggest that there are aspects of being pregnant that might remain intact and function as protective factors for mothers with a previous cancer diagnosis. However, these aspects need further exploration through longitudinal research employing larger samples and different tools which might be more accurate than self-report measures. On the other hand, our results on the data collected postnatally seems to indicate that there are aspects, such as difficulties in the interaction with their infants, which could be targeted by interventions aimed to support these mothers in their transition to parenthood. This is particularly important to prevent future child negative outcomes. Further longitudinal research is needed to better characterise difficulties experienced by mothers with cancer history and their impact on children development and to design preventive and supportive programmes for these mothers and their infants to ensure an optimal parenting environment in which children can psychologically thrive.

### Conclusion

Very little research is available on the transition to parenthood and the quality of early mother–infant interactions in samples of woman with past cancer diagnosis compared to women without. This observational and longitudinal pilot study shed light on the quality of prenatal attachment, women’s psychological well-being in pregnancy and characteristics of the interactions between mothers with past cancer and their infants, fundamental influencing factors for later child development. More research is needed to deepen the understanding of risk and protective factors experienced by these women to effectively support them in the transition to motherhood and to consequently prevent the negative effects of maternal difficulties in establishing a relationship with their infants on later child development.

## Data Availability Statement

The raw data supporting the conclusions of this article will be made available by the authors, without undue reservation.

## Ethics Statement

The studies involving human participants were reviewed and approved by ASST Bergamo Est Hospital, IEO and IRCCS “San Matteo” Hospital (196/2016). The patients/participants provided their written informed consent to participate in this study.

## Author Contributions

LBon and LBoz wrote the first draft of the manuscript. LP organized the database and performed the statistical analysis. All authors contributed to conception and design of the study, manuscript revision, read, and approved the submitted version.

## Conflict of Interest

The authors declare that the research was conducted in the absence of any commercial or financial relationships that could be construed as a potential conflict of interest.

## Publisher’s Note

All claims expressed in this article are solely those of the authors and do not necessarily represent those of their affiliated organizations, or those of the publisher, the editors and the reviewers. Any product that may be evaluated in this article, or claim that may be made by its manufacturer, is not guaranteed or endorsed by the publisher.

## References

[B1] Bantema-Joppe E. De Bock G. Woltman-van Iersel M. Busz D. Ranchor A. Langendijk J. (2015). The impact of age on changes in quality of life among breast cancer survivors treated with breast-conserving surgery and radiotherapy. *Br. J. Cancer* 112 636–643. 10.1038/bjc.2014.632 25602967 PMC4333491

[B2] Bradley S. Rose S. Lutgendorf S. Costanzo E. Anderson B. (2006). Quality of life and mental health in cervical and endometrial cancer survivors. *Gynecol. Oncol.* 100 479–486. 10.1016/j.ygyno.2005.08.023 16185753

[B3] Britto P. R. Lye S. J. Proulx K. Yousafzai A. K. Matthews S. G. Vaivada T. (2017). Nurturing care: promoting early childhood development. *Lancet* 389 91–102. 10.1016/S0140-6736(16)31390-3 27717615

[B4] Carreira H. Williams R. Dempsey H. Stanway S. Smeeth L. Bhaskaran K. (2021). Quality of life and mental health in breast cancer survivors compared with non-cancer controls: A study of patient-reported outcomes in the United Kingdom. *J. Cancer Surviv.* 15 564–575. 10.1007/s11764-020-00950-3 33089480 PMC8272697

[B5] Costa R. Figueiredo B. (2012). Infants’ behavioral and physiological profile and mother–infant interaction. *Int. J. Behav. Dev.* 36 205–214. 10.1177/0165025411428248

[B6] Deans C. L. (2020). Maternal sensitivity, its relationship with child outcomes, and interventions that address it: a systematic literature review. *Early Child Dev. Care* 190 252–275. 10.1080/03004430.2018.1465415

[B7] Della Vedova A. M. D. Dabrassi F. Imbasciati A. (2008). Assessing prenatal attachment in a sample of Italian women. *J. Reprod. Infant Psychol.* 26 86–98. 10.1080/02646830701805349

[B8] Faccio F. Mascheroni E. Ionio C. Pravettoni G. Alessandro Peccatori F. Pisoni C. (2020). Motherhood during or after breast cancer diagnosis: a qualitative study. *Eur. J. Cancer Care* 29:e13214. 10.1111/ecc.13214 31904906

[B9] Farnè M. Sebellico A. Gnugnoli D. Coralli A. (1991). *POMS. Profile of Mood States: Adattamento Italiano.* Firenze: Organizzazioni Speciali.

[B10] Gunning M. Conroy S. Valoriani V. Figueiredo B. Kammerer M. H. Muzik M. (2004). Measurement of mother-infant interactions and the home environment in a European setting: preliminary results from a cross-cultural study. *Br. J. Psychiatry* 184 S38–S44. 10.1192/bjp.184.46.s38 14754817

[B11] Hazell Raine K. Nath S. Howard L. M. Cockshaw W. Boyce P. Sawyer E. (2020). Associations between prenatal maternal mental health indices and mother–infant relationship quality 6 to 18 months’ postpartum: A systematic review. *Infant Mental Health J.* 41 24–39. 10.1002/imhj.21825 31524300

[B12] Heinisch C. Galeris M.-G. Gabler S. Simen S. Junge-Hoffmeister J. Fößel J. (2019). Mothers with postpartum psychiatric disorders: proposal for an adapted method to assess maternal sensitivity in interaction with the child. *Front. Psychiatry* 10:471. 10.3389/fpsyt.2019.00471 31396110 PMC6661973

[B13] Lefkovics E. Baji I. Rigó J. (2014). Impact of maternal depression on pregnancies and on early attachment. *Infant Mental Health J.* 35 354–365. 10.1002/imhj.21450 25798487

[B14] Mascheroni E. Faccio F. Bonassi L. Ionio C. Peccatori F. A. Pisoni C. (2020). Exploring differences in psychological aspects during pregnancy between cancer survivors and women without a history of cancer. *Support. Care Cancer* 28 2255–2263. 10.1007/s00520-019-05048-w 31463591

[B15] Mitchell A. J. Ferguson D. W. Gill J. Paul J. Symonds P. (2013). Depression and anxiety in long-term cancer survivors compared with spouses and healthy controls: a systematic review and meta-analysis. *lancet Oncol.* 14 721–732. 10.1016/S1470-2045(13)70244-4 23759376

[B16] Murray L. Fiori-Cowley A. Hooper R. Cooper P. (1996). The impact of postnatal depression and associated adversity on early mother-infant interactions and later infant outcome. *Child Dev.* 67 2512–2526. 10.1111/j.1467-8624.1996.tb01871.x 9022253

[B17] Petersen R. Petermann F. Petermann U. (2017). Sensitive Parenting and Emotion Regulation in Children: A Systematic Review. *Kindheit Und Entwicklung* 26 147–156.

[B18] Rocha N. A. C. F. dos Santos Silva F. P. Dos Santos M. M. Dusing S. C. (2020). Impact of mother–infant interaction on development during the first year of life: A systematic review. *J. Child Health Care* 24 365–385. 10.1177/1367493519864742 31337225

[B19] Rogers A. Obst S. Teague S. J. Rossen L. Spry E. A. Macdonald J. A. (2020). Association between maternal perinatal depression and anxiety and child and adolescent development: a meta-analysis. *JAMA Pediatrics* 174 1082–1092. 10.1001/jamapediatrics.2020.2910 32926075 PMC7490743

[B20] Schmitt F. Jyrkkiö S. Tamminen T. Piha J. (2010). Cancer during pregnancy: two case studies. *Infant Mental Health J.* 31 71–93. 10.1002/imhj.20243 28543590

[B21] Shin H. Park Y. J. Kim M. J. (2006). Predictors of maternal sensitivity during the early postpartum period. *J. Adv. Nurs.* 55 425–434. 10.1111/j.1365-2648.2006.03943.x 16866838

[B22] Stover A. M. Mayer D. K. Muss H. Wheeler S. B. Lyons J. C. Reeve B. B. (2014). Quality of life changes during the pre-to postdiagnosis period and treatment-related recovery time in older women with breast cancer. *Cancer* 120 1881–1889. 10.1002/cncr.28649 24647996 PMC4047201

[B23] Vandenbroucke T. Verheecke M. van Gerwen M. Van Calsteren K. Halaska M. J. Fumagalli M. (2020). Child development at 6 years after maternal cancer diagnosis and treatment during pregnancy. *Eur. J. Cancer* 138 57–67. 10.1016/j.ejca.2020.07.004 32858478 PMC7532701

